# Supplementary material for: Measures to assess quality of postnatal care: A scoping review
Source: PLOS Glob Public Health. 2023 Feb 17;3(2):e0001384. doi: 10.1371/journal.pgph.0001384 (PMC10021656; doi:10.1371/journal.pgph.0001384)
Supplement: Attachment — Submitted filename: ReplyPNCpaper.docx [file pgph.0001384.s004.docx]

Dear Reviewer and Editor,

Thank you for carefully reading the manuscript and constructive comments. We took time to review every detail and improve according to your comments. All comments have been addressed and changes to the manuscript have been saved under track changes. We are grateful to the reviewer and editor for their time and helpful comments.

Review Comments to the Author

Reviewer #1: Thank you for the opportunity to review this excellent manuscript. It is well written, timely, informative and the methods are well documented. I only have minor comments, mostly related to the phrasing/naming of key concepts.
 **1. The authors refer to the 2022 WHO PNC recommendations, but they also use the term “PNC guideline” to refer to this document. Is this a correct and accepted synonym? They also use the word “recommendation” to mean an individual element of care which is listed in the document, so maybe you decided to use the word “guideline” to differentiate these two concepts? Please can you explain in 1 sentence? The document is already heavy on terms and clarity can be improved.**

We acknowledge some concepts and terms should have been defined better, especially for a complex topic such as quality of care. Indeed guideline refers to the complete document (including more than just recommendations, e.g. the definition of a positive postnatal experience) while the recommendations refer to the specific listed recommendations. We have added clarification in the background:

*P4 line 35: In March 2022, the World Health Organization (WHO) published a new guideline entitled “WHO recommendations on maternal and newborn care for a positive postnatal experience”, to improve the quality of postnatal care – including provision and experience – for women and newborns with the ultimate goal of improving their health and well-being.* *Within the guideline, a positive postnatal experience is defined as “an experience in which women, newborns, partners, parents, caregivers and families receive information, reassurance and support in a consistent manner from motivated health workers; where a resourced and flexible health system recognizes the needs of women and babies and respects their cultural context” (7). The WHO PNC recommendations in the guideline address clinical and non-clinical maternal and newborn care, health promotion and health systems interventions during the six-week period (42 days) after birth* *(1). It updates and expands upon the 2014 PNC recommendations and complements WHO existing guidelines on the management of postnatal complications (7). The recommendations have a wide scope, are for all settings and include aspects such as early child development and mother-infant bonding.The 2022 WHO PNC guideline proposes that implementation and impact of these recommendations should be monitored at facility, sub-national and national levels. Monitoring should be based on clearly defined criteria and with measures that are associated with locally agreed targets.*

In addition, we have improved consistency throughout the text by always referring to the 2022 WHO PNC recommendations (if we refer to the specific listed recommendations) and guideline if we refer to the complete document.

**2. At the end of the Introduction section (as well as in the title), the focus of the study is on “quality of postnatal care”. I was really excited about this clear focus, and thought that it would be clearly differentiated from coverage. However, there was no information provided in the Introduction about how the authors defined “quality”. This would be really helpful because many (including me) struggle when reviewing literature to separate various concepts of coverage, content, contact and quality – it would be helpful to the reader to follow how the authors approached this given they are looking at a fairly large body of literature. It might also help to refer here to widely accepted frameworks, such as the WHO maternal healthcare quality framework – it can be helpful for synthesis in Results and Discussion. It is mentioned on page 7, but only in terms of helping identify search terms, not as an analytical framework or a way to help synthesise findings and identify gaps**

We indeed relied on the WHO maternal healthcare quality framework, but have not emphasized this enough throughout the paper.

We have added the framework in the background section for clarification:

*P4 line 51: WHO has placed provision and experience of care are at the core of the WHO framework for improving the quality of care for mothers and newborns around the time of childbirth, including the postnatal period (8). Within this framework, outcomes include coverage of key practices and people centred outcomes.*

To be more consistent and clarify we use this framework throughout the paper we have also explicitly mentioned this again in the methods under in- and exclusion criteria (p6 line 97):

*Inclusion criteria:*

*-Describing quality of PNC, including provision or experience of care.*

*Exclusion criteria:*

*-Articles only reporting on PNC uptake (Yes/No) in one indicator, without including any measurement of provision or experience of care. Reporting on the actual content (=provision) or experience of PNC was a requirement for inclusion.*

Lastly, we have added the encountered challenges with defining quality of care under the discussion (p19, line 309):

*Several studies within our review used a limited set of measures (e.g., the percentage of women and newborn with a postnatal check-up within two days or the percentage of women being satisfied with care) to assess PNC “quality”. Some measures were also poorly defined, leaving doubt on what care was actually provided. For this reason, “uptake of PNC” was not considered as a measure for quality of postnatal care within our review, because uptake alone does not reveal actual provision (what care was provided) or experience of care as outlined in the WHO quality of care framework (8). Further efforts among researchers to be transparent and distinguish the different concepts of uptake, coverage, content and the umbrella term “quality of care” are needed to improve comparability of data across different studies and settings. Only a minority of resources provided measures for a broader understanding of quality of care, including both provision of and experience of care (37). Recognition of the multifaceted nature of quality of care is critical for prioritising health interventions within postnatal care and increasing uptake of services along the continuum (101).*

**3. To continue with the point above, in the Methods section -> Inclusion criteria, the key one is related to “Describing quality of PNC, including provision and experience of care”. This is quite broad and fairly generic. I am none the wiser about how the author team made the “call” about including or excluding papers. What is a “quality of PNC provision” indicator? Can you give examples? For example, page 18 line 287 – you talk about percentage of women with PNC check within 2 days – this sounds to me like a measure of coverage, not a measure of quality. How did you draw the lines? Especially as “PNC uptake” alone was specifically excluded. Easiest would be to link to the WHO QoC framework if this were to be included in the Introduction, but this is just a suggestion. Did you as an author team have problems/challenges with differentiating uptake indicators from quality of provision indicators? If so, would you mind sharing this briefly with the readers (in the Discussion section, for example). This can be very insightful.**

Indeed we had challenges to draw the line but agreed within the team that uptake alone cannot be considered as a measurement of quality of care, if it is unclear what was actually provided (=content of care/provision). We have clarified and emphasized throughout the paper (see responses above).

If studies reported there was a PNC check, without including any details about the actual content of this PNC check (e.g. physical examination of the baby/mother, breastfeeding counselling, vaccination…) this study was excluded. If there was more information (including additional indicators/information such as which examinations were performed, , …) we included the article and reported measures. This was also added in the discussion (see responses to point 2 above).

**4. Going further on the point of quality, once the inclusion/exclusion criteria were explained, the manuscript hardly ever refers back to the key focus of the paper on “quality of PNC” – it uses much a more generic term “measures”. I would strongly urge the authors to keep repeating “PNC quality of care measures” to help readers stay focused on this point. Otherwise, it becomes very easy to drift off and think of all “PNC indicators” or “PNC measures”. One example is the title of Table 2 – the word quality needs to be included. Thank you for providing your clear explanation for the choice of the word measure as opposed to indicator.**

Thank you for this important point. We have added the focus on quality of PNC measures throughout the manuscript to be more consistent and clearer. In the abstract, introduction, methods, results and discussion we have improved the terminology and consistency (see track changes).

**5. Figure 1. The word recommendation is misspelled. You clearly show the structure of the PNC recommendations. I would like to suggest that you add the two dimensions along which you are reporting the findings: provision and experience, in this visual, for ease of orientation in the Results.**

Thank you for this valuable suggestion. We have added the two dimensions in the visual and corrected the typo.

**6. Page 11, line 184. Suddenly the term “resource(s)” starts to be used here. What does this mean? Do you mean it in terms of “an included document” (whether a published paper or a report)? Or is it a synonym for “measures”? I was confused in this paragraph, for example: “Number of resources per recommendation….” And then “the following recommendations for the care of women had no measures”. Are you matching the various measures to each PNC recommendation or the number of documents? Or is a resource a synonym for measure? Can one resource have multiple measures (in a sense that one paper can propose more than one indicator)? Please also look at last column in Table 2 – should this be number of resources or number of measures?**

We acknowledge these words might have caused confusion and have used other terms and clarified this now in the methods (p8, line 150):

*In this manuscript we refer to the studies (including scientific studies and grey literature reports) with identified measures, while the additional file (S3 Table) gives an overview of all identified measures per study.*

We also added this in the results for clarification (p12 line 192):

*Number of studies (including scientific studies and reports from grey literature) per recommendation varied from 0-18.*

**7. As a reader, I would have welcomed more concrete examples of the measures you identified in the main text, because until the Discussion section (line 287), there are no examples of the actual measures extracted from the included papers, all we are seeing is the numbers of measures which are mapping onto the various recommended elements of care. I know all this is in the Supplementary file 3. This is a discretionary comment for the authors to consider.**

We have chosen not to list the measures in the results section because they are just too numerous and often varying slightly in definitions, instead we refer to the supplementary file for a full overview. This was done to avoid we give the impression to prioritize some measures/select certain measures over others or list some as “standard”, which was not the aim of this review. We recommend prioritization of certain measures is the next step. However, we have tried to discuss the problem of the wide range of measures (mostly not validated), with examples, in the section “Gaps in measurement”. We hope this explanation clarifies why we did not add examples of measures in the manuscript itself.

**8. Were there measures of PNC quality that you found which did not map onto any of the PNC recommendations/elements of care? Maybe I missed this, but would be good to see.**

We originally removed this from results because they could not be mapped for logical reasons (other focus/scope) but acknowledge this is important for the readers. We have added these in S3 Table (tab 2) and in results (p17, line 259)

*Measures not mapped to the WHO PNC recommendations*

*PNC quality of care measures not mapped to the recommendations can be found in an additional file (****S3 Table****). These included quality of care measures for care immediately after childbirth, which is not addressed in the 2022 WHO PNC guideline but in the WHO guideline “Intrapartum care for a positive childbirth experience”. Also specific quality of care measures for small or sick newborns could not be mapped to the recommendations, since the guideline is targeting routine care for healthy newborns and women after childbirth. Other measures were often very (context) specific (e.g. “did you receive a hot drink”), making it impossible to map them to 2022 WHO PNC recommendations.*

**Minor points:
1. Page 10, line 168: 32% of studies are not “most”, this means more than half. Suggest rephrase to “most common”
2. Page 7, line 135. Funding resources – I think you mean funding sources?
3. Page 18, line 297 – lacking behind or lagging behind?
4. In Supplementary material 3, you use “indicator” not “measure”, this is inconsistent with the main text.**

Thank you, we have included the suggested edits.

With best wishes,

Anna Galle on behalf of the authors.

PhD

Ghent University
